# A New Polyoxygenated Flavonol Gossypetin-3-*O*-*β*-d-Robinobioside from *Caesalpinia gilliesii* (Hook.) D. Dietr. and In Vivo Hepatoprotective, Anti-Inflammatory, and Anti-Ulcer Activities of the Leaf Methanol Extract

**DOI:** 10.3390/molecules24010138

**Published:** 2018-12-31

**Authors:** Mahmoud Emam, Mohamed A. El Raey, Alaadin E. El-Haddad, Sally A. El Awdan, Abdel-Gawad M. Rabie, Mohamed A. El-Ansari, Mansour Sobeh, Samir M. Osman, Michael Wink

**Affiliations:** 1 Department of Phytochemistry and Plant Systematics, National Research Centre, Dokki, Cairo 12622, Egypt; mahmoudemamhegazy2020@gmail.com (M.E.); ansarialaa@hotmail.com (M.A.E.-A.); 2 Department of Pharmacognosy, Faculty of Pharmacy, October 6 University, Cairo 12566, Egypt; alaa_elhaddad.ph@o6u.edu; 3 Department of Pharmacology, National Research Centre, Dokki, Cairo 12622, Egypt; sallyelawdan@ymail.com; 4 Department of Chemistry, Faculty of Science, Ain Shams University, Abbassia, Cairo 11566, Egypt; Mohamed.abdelgawad@hotmail.com; 5 Department of Pharmaceutical Biology, Institute of Pharmacy and Molecular Biotechnology, Heidelberg University, Im Neuenheimer Feld 364, 69120 Heidelberg, Germany; sobeh@uni-heidelberg.de

**Keywords:** *Caesalpinia gilliesii*, flavonoids, anti-inflammatory, anti-ulcer, hepatoprotective activities

## Abstract

A hitherto unknown polyoxygenated flavonol robinobioside (gossypetin-3-*O*-*β*-d-robinobioside) was isolated from the leaves of *Caesalpinia gilliesii* along with thirteen known phenolic secondary metabolites. The isolated compounds were characterized using spectroscopic analysis, including 1D and 2D NMR and mass spectrometry (MS) analyses. The extract reduced the level of liver damage in CCl_4_-induced liver injury in rats. A decrease of the liver biomarkers—aspartate aminotransferase (AST) and alanine aminotransferase (ALT) and an increase of total antioxidant capacity (TAC) levels—were observed similar to the liver protecting drug silymarin. In addition, the extract showed promising activity against carrageenan-induced paw edema in rats and protected their stomachs against ethanol-induced gastric ulcers in a concentration dependent fashion. The observed activities could be attributed to the high content of antioxidant polyphenols. Our results suggest that the *C. gilliesii* has the capacity to scavenge free radicals and can protect against oxidative stress, and liver and stomach injury.

## 1. Introduction

Drug-induced liver injury is still a major challenge. Environmental toxicants, ingested metals, and some orally consumed drugs are renown causes of liver injury [1]. For instance, the uncontrolled use of non-steroidal anti-inflammatory drugs for the treatment of pain and inflammation carries the risk of liver toxicity as well as other serious adverse effects, such as peptic ulcers and gastrointestinal bleeding [2,3,4]. Thus, medicinal therapy is still in need for novel multi-functional approaches to tackle health problems and avoid or reduce adverse drug effects.

In this regard, plant secondary metabolites offer an interesting potential for pharmacological applications where they can serve as lead drugs in clinical trials for the treatment of various diseases [5]. The genus *Caesalpinia*, a member of the family Fabaceae (subfamily Caesalpinioideae) (148 genera, ca. 4400 species), comprises more than 500 species of tropical and subtropical trees and shrubs [6]. Many species of the genus *Caesalpinia* exhibit interesting biological activities and thus are used in folk medicine as antioxidant, anti-inflammatory, antimicrobial, anticancer, antidiabetic, hepatoprotective, and antiviral agents [7,8,9,10]. Plants of this genus produce flavonoids, steroids, triterpenoids, tannins, saponins, alkaloids, and terpenes [7,8,9,10].

The Yellow Bird of Paradise, *Caesalpinia gilliesii* (Hook.) D. Dietr, is native to Argentina and now is presently cultivated worldwide in the tropics and subtropics [6,11]. Recently, phenolics, fatty acids, and phytosterols were isolated from the flowers of *C. gilliesii* and the extract exhibited cytotoxic and hepatoprotective activities [11]. Moreover, the alcoholic extract of *C. gilliesii* leaves demonstrated substantial free radical scavenging activities in vitro [12].

In the present study, the hepatoprotective, anti-inflammatory, and anti-ulcer activities of the methanol extract from *C. gilliesii* leaves were evaluated in animal models. Further chromatographic analysis has led to the isolation of fourteen polyphenols, including one hitherto unknown, identified as gossypetin-3-*O*-*β*-d-robinobioside. The structure elucidation of all isolated compounds was done using spectroscopic analysis, including 1D and 2D NMR spectroscopy and mass spectrometry.

## 2. Results

### 2.1. Phytochemical Composition

The phytochemical analysis of the methanol extract from *C. gilliesii* leaves revealed a new flavonol robinobioside, namely gossypetin-3-*O*-*β*-d-robinobioside (**11**) together with thirteen known phenolic secondary metabolites, namely quercetin (**1**), quercetin-3-*O*-*β*-d-glucoside (**2**), quercetin-3-*O*-*β*-d-galactoside (**3**), rutin (**4**), quercetin-3-*O*-*β*-d-robinoside (**5**), kaempferol-3-*O*-*β*-d-rutinoside (**6**), luteolin (**7**), luteolin-7-*O*-*β*-d-glucoside (**8**), isorhamnetin (**9**) [13,14,15,16], gossypetin-3-O-*β*-d-rutinoside (**10**) [16,17,18,19], *p*-hydroxybenzoic acid (**12**) [20], gallic acid (**13**) [21], and brevifolin carboxylic acid (**14**) [22]. The NMR data of compounds (**1**–**10**, **12**–**14**) are shown in a supplementary file.

**Figure 1:**
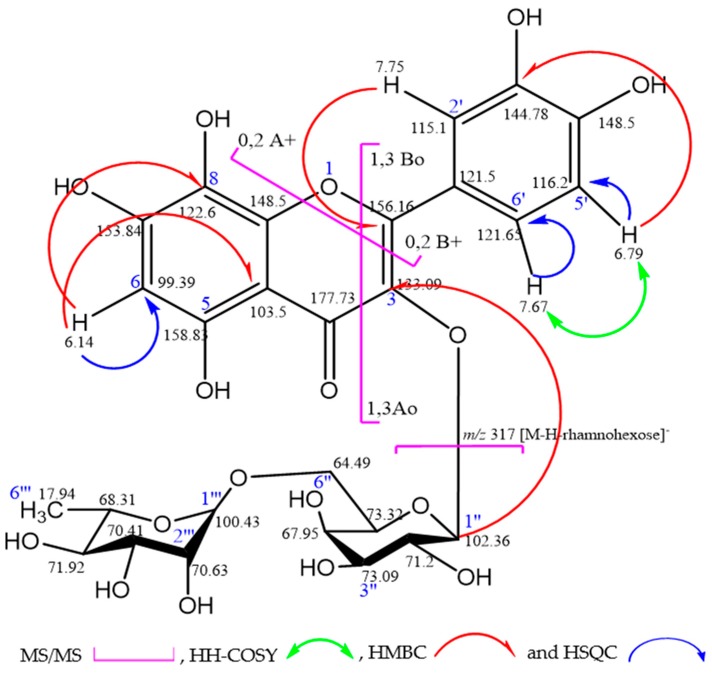
Selected fragmentation pattern (MS/MS) and correlations of gossypetin-3-*O*-*β*-d-robinobioside.

Compound (**11**) appeared as a yellow amorphous powder. In paper chromatography, the dark purple spot changed into a yellow color when exposed to ammonia vapor. The UV spectral data of this compound exhibited pronounced major absorption bands at λ_max_ = 352 and 261 nm, corresponding to band I and band II of flavonoids, respectively, which confirmed the highly oxygenated flavonol nature with 3-hydroxyl substituent [13]. Moreover, MS/MS spectrum showed a quasi-molecular ion [M − H]^−^ at *m*/*z* 625, and different fragments at *m/z* 317 [M − H − rhamnohexose]^−^, 457 [M − H − rhamnohexose − (1,3A_o_ − B_o_)]^−^, and 489 [M − H − (0,2A^+^ − B^+^)]^−^, as shown in Figure 1. In addition, the ^1^H-NMR spectrum displayed aromatic proton signals, appearing as doublet at δ 7.76 ppm corresponding to H-2′ and doublet of doublet at 7.67 ppm to H-6′ that was stereo chemically *meta*-coupled with H-2′ and *ortho*-coupled with H-5′, which appeared as doublet signal with *J* = 8.5 Hz at δ 6.79 ppm. The compound had C-8 substitution indicated by the disappearance of H-8 signal and the appearance of singlet signal at δ 6.14 corresponding to H-6. The attached proton test (APT NMR) showed different chemical shifts of carbon signals such as C-4 at δ 177.76, oxygenated *Sp*^2^ carbons C-2, C-3, C-5, C-7, C-8, C-9, C-3′, and C-4′ at δ 156.16, 133.09, 158.83, 153.8, 122.6, 148.47, 144.78, and 148.42, respectively. The downfield shift of C-8 signal to 122.6 ppm indicated that the substitution was at position-8. Moreover, the upfield shift of C-3 signal at δ 133.09 ppm confirmed the glycosidic linkage with aglycone at position C-3. On the other hand, *Sp*^2^ carbon signals appeared at δ 98.39, 103.5, 121.53, 115.1, 116.2, and 121.65 assigned for C-6, C-10, C-1′, C-2′, C-5′, and C-6′, respectively. The structure of the aglycone was deduced to be gossypetin in agreement with previous published data [16,17,18]. Two anomeric sugar protons were also observed in ^1^H-NMR spectrum at δ 5.35 (d, *J* = 7.7 Hz, H-1″) and 4.45 (br. s, H-1‴), indicating that compound (**11**) is a diglycoside. One sugar unit was elucidated as terminal rhamnose unit confirmed by H-1‴ broad singlet at δ 4.45 and the doublet signal of the methyl protons of rhamnose (3H-6‴) with *J* = 6.2 Hz at δ 1.08. The rhamnose unit was directly attached to the hexose sugar unit.

The anomeric carbon signals of di-sugars, appearing at δ 102.36 and 100.43, were assigned to C-1″ hexose and C-1‴ rhamnose, respectively. The other carbon signals of hexose sugar could reveal a galactoside (Table 1) in agreement with reported data [16,19]. To confirm the nature of the sugar moieties, the compound was subjected to complete acid hydrolysis with 2 N HCl for 3 h and the hydrolysis products were identified on paper chromatographyas galactose and rhamnose when compared to authentic sugar samples [13].

The structure confirmation of compound (**11**) was achieved by 2D NMR spectroscopy, including an HH COSY (homonuclear correlated spectroscopy) experiment that showed cross peaks for the adjacent protons (neighboring protons), an HSQC (heteronuclear single quantum coherence spectroscopy) experiment that showed direct attachment between the proton and its carbon, and the interglycosidic linkage by HMBC (heteronuclear multiple bond correlation) (Figure 1).

Therefore, the compound was identified as gossypetin-3-*O*-*β*-d-robinobioside [rhamnosyl (1→6) galactoside] with a molecular formula of C_27_H_30_O_17_. This is the first report of the isolation and identification of such compound in nature. The spectral data of the compound are shown in the supplementary file (Figures S1–S6).

### 2.2. Hepatoprotective Activity

**Figure 2:**
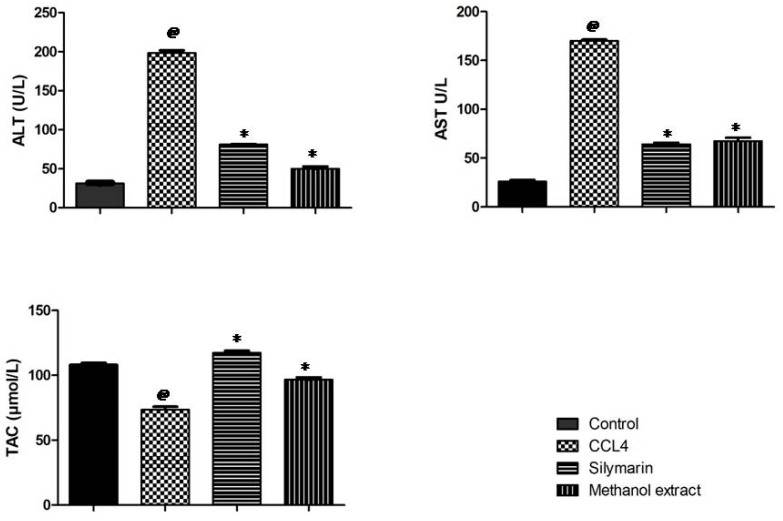
Hepatoprotective effect of *Caesalpinia gilliesii* leaves on liver biomarkers in CCl_4_-induced hepatotoxicity in rats (*n* = 6). ^@^ *p* < 0.01 vs control group. * *p* < 0.01 vs CCl_4_ group.

After CCL_4_ injection (on day 8), a significant elevation in the liver biomarkers aspartate aminotransferase (AST)(U/L) and alanine aminotransferase (ALT)(U/L) was observed in the CCL_4_ group compared to the control group. Rats pretreated with the silymarin (a flavonolignan from *Silybum marianum*) were apparently protected against liver injury when compared to the CCl_4_ group (*p* < 0.01) (Figure 2). Also, CCl_4_ administration led to a significant decrease (*p* < 0.01) in TAC (μmole/L) compared to the control group. Pretreatment of rats with the methanol extract significantly increased (*p* < 0.01) the level of total antioxidant capacity (TAC) compared to the CCl_4_ treated group. Also, pretreatment with the methanol extract resulted in a significant decrease in the enzyme levels, which indicates a protection of hepatic tissue damage caused by CCl_4_ (Figure 2). The significant hepatoprotective activity of the methanol extract may be attributed to its high content of polyphenols and their antioxidant activities [12].

### 2.3. Histopathological Investigation

**Figure 3:**
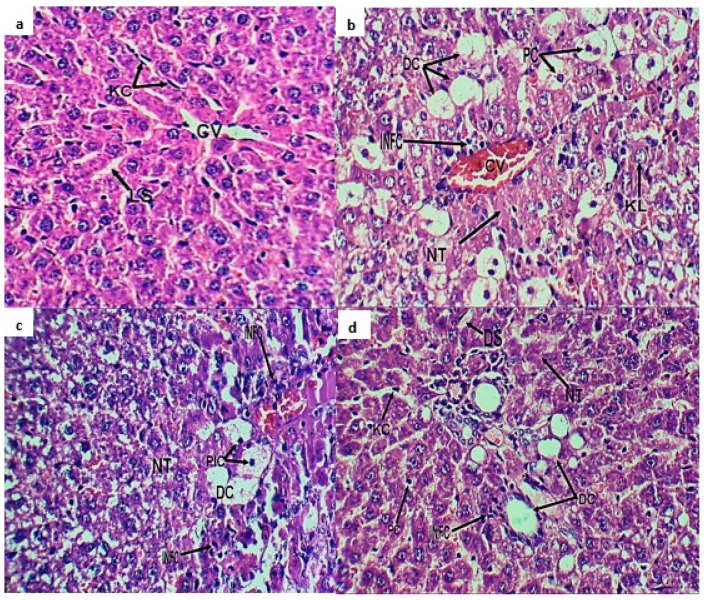
Photomicrograph of liver sections (**a**) normal liver with normal hepatocytes, normal hepatic cords and normal distribution of Kupffer cells (KC) with normal liver sinusoids (LS); (**b**) CCL_4_ group showed marked infiltration of inflammatory cells (INF), marked degenerative changes, necrotic tissue (NT), and degenerated cells (DC) with clear pyknotic reaction (PC); (**c**) silymarin group showed moderate hepatocyte degeneration; (**d**) methanol extract group exhibited mild nuclear changes and apoptosis, mild necrotic changes, normal appearance of Kupffer cells, and mild dilatation of liver sinusoids with normal tissue histological-architecture. Central vein (CV), infiltration of inflammatory cells (INFC), karyolysis (KL), pyknotic changes (PIC), and dilated liver sinusoids (DS).

Histopathological observations on organ morphology confirmed the biochemical analyses. Normal hepatocytes are shown in Figure 3a. In the CCL_4_ group, the liver sections showed a loss of cellular architecture, marked degenerative changes, enlarged nuclei, cell infiltration with clear pyknotic reaction, and dilated liver sinusoids, as well as other nuclear changes (karyolysis and karyorrhexis) (Figure 3b). Silymarin pretreatment resulted in moderate cellular degeneration with more or less normal hepatocytes (Figure 3c). Rats pretreated with methanol extract showed mild nuclear changes and apoptosis, mild necrotic changes and normal appearance of Kupffer cells, and mild dilatation of liver sinusoids with normal tissue histological-architecture (Figure 3d).

### 2.4. Anti-Inflammatory Activity: Carrageenan-Induced Edema Assay

**Figure 4:**
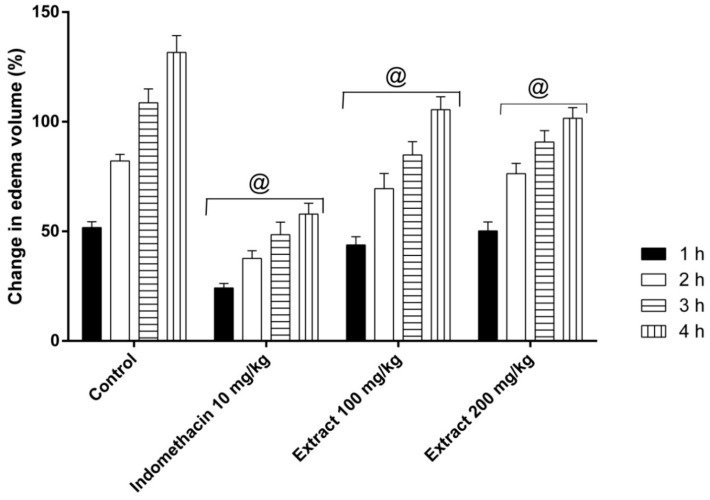
Anti-inflammatory protective effect of *C. gilliesii* leaves on carrageenan-induced inflammation in rats. The data are represented as means ± SE. ^@^ *p* < 0.05 vs control group.

The carrageenan injection resulted invisible redness and pronounced edema in the hind paws of rats after 4 h [23]. Indomethacin, a well-known non-steroidal anti-inflammatory drug, significantly decreased carrageenan-induced edema (in agreement with many studies) [24]. Oral administrations of the methanol extract (100 and 200 mg/kg) one hour before induction of inflammation significantly (*p* < 0.05) reduced the edema volume at all-time points (Figure 4).

### 2.5. Anti-Ulcer Activity

**Figure 5:**
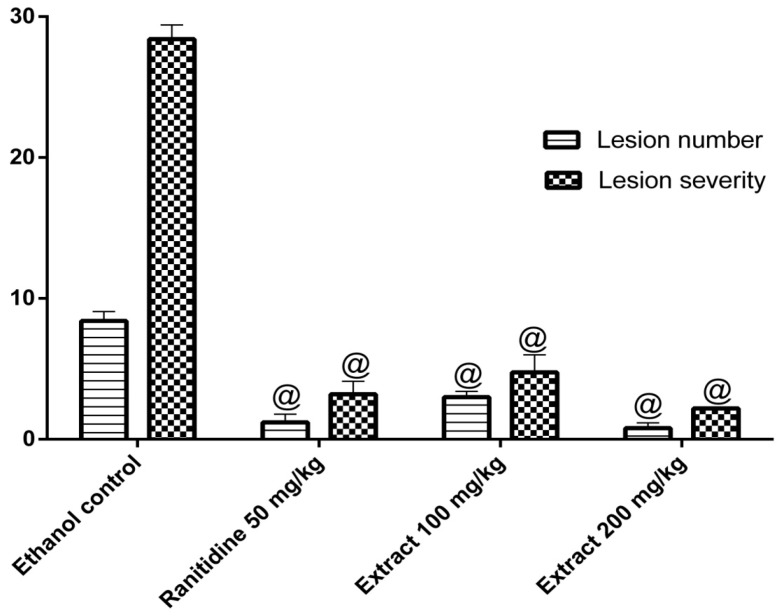
Anti-ulcer effects of the methanol extract of *C. gilliesii* leaves (*n* = 6). Statistical analysis was performed by Kruskal-Wallis non-parametric one way ANOVA followed by Mann Whitney multiple comparisons test. ^@^ *p* < 0.05 vs control ethanol group.

The oral administration of ethanol increases the acid content in stomach, which in turn may induce acute gastric lesions. The ethanol model has been widely used to explore the gastro protective effects [25,26]. Pretreatment with the methanol extract (100 and 200 mg/kg) decreased the number of lesions and reduced ulcer severity values (*p* < 0.05) compared to the control ethanol group (Figure 5). These results can probably be attributed to the inhibition of gastric acid secretion or the inhibition of lipid peroxidation by the antioxidant phenolics in the extract [12].

## 3. Discussion

In this study, 14 phenolic secondary metabolites were isolated from the methanol extract of *C. gilliesii* leaves by column chromatography and characterized using conventional and spectroscopic analyses. Out of the isolated compounds, a new gossypetin-3-*O*-*β*-d-robinobioside [rhamnosyl (1→6) galactoside] was characterized. Other compounds such as quercetin and rutin [27,28], luteolin [29], isorhamnetin [30], gallic acid [28], and brevifolin carboxylic acid [21] were previously described in the genus. On the other hand, *p*-hydroxybenzoic acid, quercetin-3-*O*-*β*-d-glucoside, quercetin-3-*O*-*β*-galactoside, quercetin-3-*O*-*β*-d-robinoside, kaempferol-3-*O*-*β*-d-rutinoside, luteolin-7-*O*-*β*-d-glucoside, and gossypetin-3-O-*β*-d-rutinoside were isolated from the genus for the first time. Based on HPLC analysis, quercetin-3-*O*-*β*-d-glucoside, quercetin-3-*O*-*β*-d-galactoside, quercetin-3-*O*-*β*-d-robinoside, kaempferol-3-*O*-*β*-d-rutinoside, luteolin-7-*O*-*β*-d-glucoside, and rutin dominated in the extract (HPLC chromatogram is shown in Figure S7).

Reactive oxygen species (ROS) are involved in the development of several health disorders, among them liver injury. CCl_4_ induces severe hepatic injury with a multivariate damage, and among them are free radicals propagations [31]. In the current study, the methanol extract counteracted the deleterious effects of CCl_4_ induced liver injury in rats (reduction of ALT, AST and increase of TAC). Also, the extract protected the liver architecture against the pathologic effects of CCl_4_ at a dose of 300 mg/kg. These activities might be attributed to the high content of polyphenolic compounds such as quercetin-3-*O*-*β*-d-glucoside, quercetin-3-*O*-*β*-d-galactoside, rutin, kaempferol-3-*O*-*β*-d-rutinoside, and luteolin-7-*O*-*β*-d-glucoside. Similar activities were reported from *C. gilliesii* flowers and other *Caesalpinia* species [11,32,33].

The acute inflammation model; carrageenan-induced paw edema, is widely used for assessing anti-inflammatory candidates [34]. Oral administration of *C. gilliesii* leaves methanol extract significantly diminished carrageenan-induced paw edema in rats in the two dose levels (100 and 200 mg/kg). Flavonoids along with their glycosides, such as kaempferol, apigenin, luteolin, myricetin, and quercetin, possess anti-inflammatory effects [35,36]. These results are in agreement with those reported from *Caesalpinia pulcherrima* and others [33,37].

Ethanol-induced ulcer is a well-defined model to evaluate the gastroprotective activities of plant extracts. Ethanol induces oxidative stress, reduces gastric blood flow, and exerts a direct toxic effect on the epithelium forming necrotic lesions [38]. The current work showed that administration of *C. gilliesii* leaves methanol extract exhibited substantial gastroprotective properties; it diminished ulcer numbers and ulcer severity in a dose-dependent fashion after ethanol challenge. These activities might be attributed to the presence of antioxidant polyphenolics. Similar activities were reported from other *Caesalpinia* species, namely *C. pulcherrima*, *C. sappan*, *C. crista,* and *C. bonduc* [32,37,38,39,40].

The hepatoprotective and anti-inflammatory activities are in agreement with those reported from extracts rich in polyphenols such as *Eremophila maculata, Syzygium jambos, Syzygium aqueum,* and *Syzygium samarangense* [31,34,41,42]. To sum up, *C. gilliesii* counteracts oxidative stress in several animal models and exhibits noticeable hepatoprotective, anti-inflammatory, and anti-ulcer activities.

## 4. Material and Methods

### 4.1. Plant Material

Leaves of *C. gilliesii* were collected in May 2015 from the Borg El Arab region, Egypt. The plant was taxonomically identified and kept under the accession number M-130 at the CAIRC Herbarium of the National Research Centre (NRC), Cairo, Egypt [43].

### 4.2. Extraction and Isolation

*C. gilliesii* leaves were washed with distilled water and then dried in shade. Dried leaves (2 kg) were powdered and macerated with 70% (*v*/*v*) aqueous methanol. After solvent evaporation under reduced pressure (Rotavapor^®^ R-300, BÜCHI, Flawil, Switzerland), a portion of the total crude extract (60 g) was suspended in 500 mL of distilled water, sonicated (30 min), and defatted using *n*-hexane (5 × 1 L). After the removal of the*n*-hexane layer (2.85 g), the extract was lyophilized and then extracted with methanol (25 g). The latter was applied to a HP-20 Diaion^®^ (Sigma Aldrich, CA, USA) column chromatography. Chromatography started with 100% H_2_O and the polarity was decreased gradually with methanol (10% up to 100%). Fractions were collected and analytical paper chromatography and two-dimensional paper chromatography were used to identify the subfractions. Further purification of the fractions over Sephadex LH-20 columns (Pharmacia, Uppsala, Sweden) (using 50% methanol and butane: water 1:1; upper layer) and preparative paper chromatography (Whatman 3MM 46 × 57 cm) were used to separate and purify the isolated compounds.

### 4.3. Experimental Analysis

NMR spectroscopy (Varian, CA, USA), 300, 400 and 500 MHz): samples (10 mg/each) were dissolved in deuterated DMSO-*d*_6_ or CD_3_OD. LC-MS/MS mass spectra were recorded on a ThermoFinnigan (Thermo Electron Corporation, Austin, TX, USA) LC system coupled with the mass spectrometer (LCQ-Duo ion trap) having a (ThermoQuest, Thermo Scientific, Waltham, MA, USA) ESI source. A Silica gel C18 reversed-phase column (Zorbax Eclipse XDB-C18, Rapid resolution, 4.6 × 150 mm, 3.5 µm, Agilent, Santa Clara, CA, USA) was utilized [44]. UV measurements: UV/vis spectra were recorded on a Shimadzu model UV-240 spectrophotometer (Shimadzu, Tokyo, Japan) at UV (λ_range_ = 240:460 nm).

Compound (**11**) was identified on the basis of 1D, 2D-NMR, and MS analysis as gossypetin-3-*O*-*β*-d-robinobioside [rhamnosyl (1→6) galactoside]: a yellow amorphous powder (22 mg) (UV λ_max_ = 261, 352 nm). ^1^H-NMR (500 MHz, DMSO-*d*_6_): δ 12.62 (1H, br.s, 5-OH), 7.75 (1H, d, *J* = 2.0Hz, H-2′), 7.67 (1H, dd, *J* = 2&8.5Hz, H-6′), 6.79 (1H, d, *J* = 8.5Hz, H-5′), 6.14 (1H, s, H-6), 5.35 (1H, d, *J* = 7.7Hz, H-1″), 4.45 (1H, br.s, H-1‴), 1.08 (3H, d, *J* = 6.2Hz, H-6‴). APT-NMR (125.721 MHz, DMSO-*d*_6_): δ 156.16 (C-2), 133.09 (C-3), 177.73 (C-4), 158.83 (C-5), 98.39 (C-6), 153.84 (C-7), 122.6 (C-8), 148.5 (C-9), 103.50 (C-10), 121.50 (C-1′), 115.10 (C-2′), 144.78 (C-3′), 148.50 (C-4′), 116.2 (C-5′), 121.65 (C-6′), 102.36 (C-1″), 71.20 (C-2″), 73.09 (C-3″), 67.95 (C-4″), 73.32 (C-5″), 64.79 (C-6″), 100.43 (C-1‴), 70.63 (C-2‴), 70.41 (C-3‴), 71.92 (C-4‴), 68.31 (C-5‴), 17.94 (C-6‴). ESI-MS/MS *m*/*z =* 625 [M − H]^−^, and different fragments at *m*/*z* 317 [M − H − rhamnohexose]^−^, 457 [M − H − rhamnohexose − (1,3A_o_ − B_o_)]^−^ and 489 [M − H − (0,2A^+^ − B^+^)]^−^.

### 4.4. Biological Experiments

#### 4.4.1. Drugs and Chemicals

Silymarin (CID Co., Giza, Egypt), carbon tetrachloride, (E. Merck (I) Ltd., Mumbai, India), olive oil, carrageenan lambda (Sigma Aldrich, Steinheim, Germany), indomethacin (Kahira, Pharmaceuticals Co. Cairo, Egypt) and ranitidine (Pharco Pharmaceuticals Co. Alexandria, Egypt) were used. TAC and kits were used for the liver biomarkers; ALT and AST were purchased from Biodiagnostics Co. (Cairo, Egypt). All other chemicals were of analytical grade.

#### 4.4.2. Animals

Mature male albino Wister rats (160 ± 10 g, 10–12 weeks age) were obtained from the National Research Centre, Giza, Egypt. Animals were acclimatized to our laboratory environment prior to the experiments for 7 days and housed in colony cages (6 rats per cage) with normal light/dark cycles at temperatures of 25 ± 2 °C and free access to standard food and water. All experimental procedures and animal care methods in this study were approved by the Ethical Committee of the National Research Centre and followed the recommendations of the National Institute of Health Guide for Care and Use of Laboratory Animals (NIH1985).

#### 4.4.3. Hepatoprotective Activity

Four groups of rats (6 rats each) were treated as follows: control group and CCL_4_ group rats were fed on vehicle; silymarin group rats received 100 mg/kg silymarin p.o. once daily; methanol extract group rats received 300 mg/kg extract p.o. once daily. Silymarin and the methanol extract were administered in the morning by the gavage method. On day 7, the rats (except control) obtained a single intraperitoneal injection of 30% CCL_4_ in olive oil (1 mL/kg). On day 8, i.e., 24 h after CCL_4_ injection, blood was collected in centrifuge tubes from the rats of all groups [45]. AST and ALT were quantified using the kits described by the manufacturers. Also, TAC was estimated by ferric reducing antioxidant power (FRAP) assay [46]. The enzyme activity was expressed as units/liter (U/L).

#### 4.4.4. Histopathological Investigation

After blood sampling, rats were sacrificed and dissected. A portion of the liver was rinsed in saline solution and quickly fixed in formalin (10%) for microscopic evaluation. The specimens were then stained with haematoxylin and eosin (H and E) and subjected to histopathological analysis [31].

#### 4.4.5. Anti-Inflammatory Activity: Carrageenan-Induced Paw Edema

Four groups of rats (6 rats each) were fasted for 12 h. The control group (I) received normal saline. Group II received the reference compound indomethacin (10 mg/kg, p.o.). Groups III and IV obtained the extract (100 and 200 mg/kg, respectively). After 1 h, inflammation was inducted into the sub-planter region of the left hind paws of the animals using a carrageenan suspension (0.1 mL of 1% *w*/*v* suspension in 0.9% saline solution) [47]. After 4 h, visible redness and pronounced swelling had developed and lasted for 48 h. Initially, the paw volume was measured before and 1, 2, 3, and 4 h after carrageenan injection using a planimeter [48]. The change in edema volume for the corresponding time was calculated based on the difference between initial and subsequent readings. Anti-inflammatory activities were determined by comparison with the control group. Edema volumes were used to calculate (%) change in edema volume by using the following formula:

% change in edema volume = (edema volume after carrageenan injection − initial volume/initial volume) × 100

#### 4.4.6. Anti-Ulcer Activity: Ethanol-Induced Gastric Ulcer Model

Rats were divided into 4 groups (*n* = 6). Group I (control group) received normal saline. Group II (ranitidine group) received ranitidine (50 mg/kg in 1% tween 80). Groups III and IV received methanol extract (100 and 200 mg/kg, respectively). Intragastric administration of absolute ethanol was used to induce acute erosion of the gastric mucosa in fasting rats [49]. After 1 h of ethanol administration, animals were sacrificed by cervical dislocation. The stomachs were then excised along the greater curvature and examined macroscopically for mucosal necrotic lesions, red streaks, and red erosions [50]. The total lesion number was counted and lesion severity was recorded [51].

## 5. Statistical Analysis

Biological data are stated as mean ± SE (*n* = 6) and analyzed statistically by one-way ANOVA followed by Tukey’s post hoc test and Student’s *t*-test, which were used to state the differences between the groups’ tests using GraphPad Prism 5.0 (GraphPad Prism Software Inc., San Diego, CA, USA). The statistical significance was considered as *p* < 0.05. Lesion numbers and severity were analyzed by Kruskal-Wallis non-parametric one-way ANOVA followed by the Mann Whitney multiple comparison test.

## 6. Conclusions

We isolated 13 known secondary metabolites, along with a new flavonoid robinobioside, gossypetin-3-*O*-*β*-d-robinobioside, from the leaf extract of *C. gilliesii.* The studied extract demonstrated substantial hepatoprotective, anti-inflammatory, and anti-ulcer activities in animals. *Caesalpinia gilliesii* is a good candidate for scavenging free radicals and counteracting oxidative stress, which can occur during liver and stomach injuries. Further studies are needed to explore the modes of action of the extract.

## Figures and Tables

**Table 1:** ^1^H and APT-NMR data and HMBC correlations for compound (**11**) **(**gossypetin-3-*O*-*β*-d-robinobioside [rhamnosyl (1→6) galactoside]) recorded in DMSO-*d*_6_; δ in ppm, *J* in Hz.

Position	APT	δ_H_ (mult, *J* [Hz])	δ_C_	HMBC (H→C)
2	C		156.16	
3	C		133.09	
4	C		177.73	
5	C		158.83	
6	CH	6.14 (1H, s, H-6)	98.39	C (8), C (10)
7	C		153.84	
8	C		122.6	
9	C		148.5	
10	C		103.50	
1′	C		121.50	
2′	CH	7.75(1H, d, *J* = 2.0Hz, H-2′)	115.10	C (2), C (4′), C (6′)
3′	C		144.78	
4′	C		148.50	
5′	CH	6.79 (1H, d, *J* = 8.5Hz, H-5′)	116.2	C (1′), C (3′)
6′	CH	7.67 (1H, dd, *J* = 2&8.5Hz, H-6′)	121.65	C (2), C (2′), C (4′)
1″	CH	5.35 (1H, d, *J* = 7.7Hz, H-1″)	102.36	
2″	CH		71.20	
3″	CH		73.09	
4″	CH		67.95	
5″	CH		73.32	
6″	CH_2_		64.79	
1‴	CH	4.45 (1H, br.s, H-1‴)	100.43	
2‴	CH		70.63	
3‴	CH		70.41	
4‴	CH		71.92	
5‴	CH		68.31	
6‴	CH_3_	1.08 (3H, d, *J* = 6.2Hz, H-6‴)	17.94	

Arbitrary atom numbering at 500 and 125 MHz, respectively.

## Supplementary Materials

The following are available online. Figure S1: UV spectrum of gossypetin-3-O-β-d-robinobioside [rhamnosyl (1→6) galactoside]; Figure S2: 1H-NMR spectrum of gossypetin-3-O-β-d-robinobioside [rhamnosyl (1→6) galactoside]; Figure S3: HH-COSY NMR spectrum of gossypetin-3-*O*-*β*-d-robinobioside [rhamnosyl (1→6) galactoside]; Figure S4: APT-NMR spectrum of gossypetin-3-*O*-*β*-d-robinobioside [rhamnosyl (1→6) galactoside]; Figure S5: HSQC-NMR spectrum of gossypetin-3-*O*-*β*-d-robinobioside [rhamnosyl (1→6) galactoside]; Figure S6: HMBC-NMR spectrum of gossypetin-3-*O*-*β*-d-robinobioside [rhamnosyl (1→6) galactoside]; Figure S7: HPLC-PDA profile of the methanol extract of *Caesalpinia gilliesii*.
