# Supplementary material for: A New Polyoxygenated Flavonol Gossypetin-3-O-β-d-Robinobioside from Caesalpinia gilliesii (Hook.) D. Dietr. and In Vivo Hepatoprotective, Anti-Inflammatory, and Anti-Ulcer Activities of the Leaf Methanol Extract
Source: Molecules. 2018 Dec 31;24(1):138. doi: 10.3390/molecules24010138 (PMC6337366; doi:10.3390/molecules24010138)
Supplement: Supplementary file 1 [file molecules-24-00138-s001.pdf]

Article

A New Polyoxygenated Flavonol Gossypetin-3-*O*-*β*-D-Robinobioside from *Caesalpinia gilliesii* (Hook.) and In Vivo Hepatoprotective, Anti-Inflammatory, and Anti-Ulcer Activities of the Leaf Methanol Extract

Mahmoud Emam,[1] Mohamed A. El Raey,[1,*] Alaadin E. El- Haddad,[2] Sally A. El Awdan,[3] Abdel-Gawad M. Rabie,[4] Mohamed A. El-Ansari,[1] Mansour Sobeh,[5] Samir M. Osman,[2,*] and Michael Wink[5,*]

1 Department of Phytochemistry and Plant Systematics, National Research Centre, Dokki, Cairo 12622, Egypt; mahmoudemamhegazy2020@gmail.com (M.E.); ansarialaa@hotmail.com (M.A.E.-A.)

2. Department of Pharmacognosy, Faculty of Pharmacy, October 6 University, Cairo 12566, Egypt; Haddad002@yahoo.com (A.E.E.H.)

3 Department of Pharmacology, National Research Centre, Dokki, Cairo 12622, Egypt; sallyelawdan@ymail.com

4 Department of Chemistry, Faculty of Science, Ain Shams University, Abbassia, Cairo 11566, Egypt; Mohamed.abdelgawad@hotmail.com

5 Department of Pharmaceutical Biology, Institute of Pharmacy and Molecular Biotechnology, Heidelberg University, Im Neuenheimer Feld 364, 69120 Heidelberg, Germany; sobeh@uni-heidelberg.de

* Corresponding authors: E-mails: ma.aziz@nrc.sci.eg (M.A.E.R.), samirosman@o6u.edu.eg (S.M.O.), wink@uni-heidelberg.de (M.W.); Tel.: + 49 62 21 - 54 48 80 (M.W.)

NMR data of the identified compounds

Quercetin (1): A yellow powder (26 mg) (UV λ_{max}=256, 368 nm).^{1}H-NMR (500 MHz, DMSO-d_6): δ 12.48 (1H, s, 5-OH), 7.67 (1H, d, *J*=2.1Hz , H-2′), 7.54 (1H, dd, *J*=8.5, 2.2Hz, H-6′), 6.88 (1H, d, *J*=8.5Hz, H-5′), 6.40 (1H, d, *J*=2Hz, H-8), 6.18 (1H, d, *J*=2Hz, H-6). APT-NMR(125 MHz, DMSO-d_6): δ 146.7 (C-2), 135.6 (C-3), 175.8 (C-4), 160.7 (C-5), 98.1 (C-6), 163.9 (C-7), 93.3 (C-8), 156.1 (C-9), 102.9 (C-10), 121.9 (C-1′), 115.0 (C-2′), 145.0 (C-3′), 147.6 (C-4′), 115.6 (C-5′), 119.9 (C-6′). ESI-MS *m/z*=301 [M–H]$^-$.

Quercetin-3-*O*-*β*-D-glucoside (2): Ayellow powder (37 mg) (UV λ_{max}=255, 356 nm). ^{1}H-NMR (500 MHz, DMSO-d_6): δ 7.59 (H, d, H-2′), 7.56 (1H, dd, *J*=2.0,7.3 Hz, H-6′), 6.85 (1H, d, *J*=8.4 Hz, H-5′), 6.39 (1H, s, H-8), 6.19 (1H, s, H-6), 5.45 (1H, d, *J*=7.3 Hz, H-1″).APT-NMR(125 MHz, DMSO-d_6): δ 156.1 (C-2), 133.3 (C-3), 177.4 (C-4), 161.2 (C-5), 99.0 (C-6), 165.3 (C-7), 93.7 (C-8), 156.5 (C-9), 103.6 (C-10), 121.1 (C-1′), 115.3 (C-2′), 144.9 (C-3′), 148.7 (C-4′), 116.3 (C-5′), 121.6 (C-6′), 101.1 (C-1″), 74.2 (C-2″), 76.6 (C-3″), 69.9 (C-4″), 77.6 (C-5″), 61.0 (C-6″). ESI-MS *m/z*=463 [M–H]$^-$.

Quercetin-3-*O*-*β*-D-galactoside (3): A yellow powder (30 mg) (UV λ_{max}=256, 355 nm). ^{1}H-NMR (500 MHz, DMSO-d_6):δ 7.66 (1H, dd, *J*=8.5, 2.0 Hz, H-6′), 7.58 (1H, d, *J*= 2.0Hz, H-2′), 6.83 (1H, d, *J*= 8.5 Hz, H-5′), 6.39 (1H, s, H-8), 6.19 (1H, s, H-6), 5.37 (1H, d, *J*= 7.4 Hz, H-1″). APT-NMR(125 MHz, DMSO-d_6): δ 156.2 (C-2), 133.5 (C-3), 177.3 (C-4), 161.1 (C-5), 99.0 (C-6), 165.4 (C-7), 93.3 (C-8), 156.4 (C-9), 103.5 (C-10), 121.0 (C-1′), 115.3 (C-2′), 144.9 (C-3′), 148.5 (C-4′), 116.0 (C-5′), 121.9 (C-6′), 102.0(C-1″), 71.3 (C-2″), 73.3 (C-3″), 67.9 (C-4″), 75.9 (C-5″), 60.2 (C-6″). ESI-MS *m/z*=463 [M–H]$^-$.

Quercetin-3-*O*-*β*-D-rutinoside (Rutin) (4): A light yellow powder (64 mg) (UV λ_{max}=256, 354 nm).^{1}H-NMR (500 MHz, DMSO-d_6) δ 7.55 (1H, d, *J*=2.1 HZ, H-2'), 7.53 (1H, dd, *J*=8.2&2.1Hz, H-6'), 6.83 (1H, d, *J*=8.2Hz, H-5'), 6.37 (1H, d, *J*=1.6Hz, H-8), 6.17 (1H, d, *J*=1.6Hz, H-6), 5.32 (1H, d, *J*=7.3Hz, H-1''), 4.38 (1H, d, *J*=1.2 Hz, H-1'''), 0.98 (3H, d, *J*=6.2Hz, H-6'''). APT-NMR (125 MHz, DMSO-d_6): δ 156.44 (C-2), 133.21 (C-3), 177.10 (C-4), 161.10 (C-5), 98.93 (C-6), 165.02(C-7), 93.71 (C-8), 156.44 (C-9), 103.55 (C-10), 120.98 (C-1'), 115.27 (C-2'), 144.83 (C-3'), 148.64 (C-4'), 116.16 (C-5'), 121.56 (C-6'), 101.31 (C-1''), 74.06 (C-2''), 76.46 (C-3''), 70.55 (C-4''), 75.85 (C-5'') 66.96 (C-6''), 100.74 (C- 1'''), 70.33 (C-2'''), 69.96 (C-3''') 71.85 (C-4'''), 68.22 (C-5'''), 17.74 (C-6'''). ESI-MS *m/z*=609 [M–H]$^-$.

Quercetin-3-*O*-*β*-D-robinobioside(5): A yellow powder (33 mg) (UV λ_{max}=254, 353 nm). ^{1}H-NMR (500 MHz, DMSO-d_6) δ 7.65 (1H, dd, *J*=8.5&2.2Hz, H-6'), 7.53 (1H, d, *J*=2.2 HZ, H-2'), 6.82 (1H, d, *J*=8.5Hz, H-5'), 6.41 (1H, d, *J*=1.8Hz, H-8), 6.19 (1H, d, *J*=1.8Hz, H-6), 5.31(1H, d, *J*=7.3Hz,H-1''), 4.42 (1H, d, *J*=1.2Hz, H1'''), 1.06 (3H, d, *J*=6.2Hz, H-6'''); APT-NMR (DMSO-d_6, 125 MHz): δ 156.38 (C-2), 133.47 (C-3), 177.38 (C-4), 161.16 (C-5), 98.71 (C-6), 164.23(C-7), 93.56 (C-8), 156.32(C-9), 103.85 (C-10), 121.03 (C-1'), 115. 21 (C-2'), 144.82 (C-3'), 148.49 (C-4'), 115.98 (C-5'), 121.89(C-6'), 102.02 (C-1''), 71.09 (C-2''), 73.07 (C-3''), 68.03 (C-4''), 73.55 (C-5'') 65.11 (C-6''), 100.00 (C- 1'''), 70.62 (C-2'''), 70.41 (C-3''') 71.93 (C-4'''), 68.26 (C-5'''), 17.92 (C-6'''). ESI-MS *m/z*=609 [M–H]$^-$.

Kaempferol-3-*O*-*β*-D-rutinoside (6): A yellow amorphous powder (42 mg) (UV λ_{max}=265, 349 nm).^{1}H-NMR (500 MHz, DMSO-d_6): δ 12.53 (1H, s, 5-OH), 7.97 (2H, d, *J* = 8.8Hz, H-2′, 6′), 6.87 (2H, d, *J*=8.8Hz, H-3′, 5′), 6.37 (1H, d, *J*=1.6Hz, H-8), 6.16 (1H, d, *J*=1.6Hz, H-6), 5.29 (1H, d, *J*=7.5 Hz, H-1″), 4.38 (1H, br. s, H-1‴), 0.98 (3H, d, J = 6.1 Hz, H-6‴). APT-NMR (125 MHz, DMSO-d6): δ 156.55 (C-2), 133.15 (C-3), 177.17 (C-4), 161.12 (C-5), 98.99 (C-6), 166.96 (C-7), 93.87 (C-8), 156.63 (C-9), 103.56 (C-10), 120.85 (C-1′), 130.81 (C-2′, 6′), 115.08 (C-3′, 5′), 159.91 (C-4′), 101.45 (C-1″), 74.17 (C-2″), 76.37 (C-3″), 70.58 (C-4″), 75.71 (C-5″), 67.40 (C-6″), 100.76 (C-1‴), 70.33 (C-2‴), 69.89 (C-3‴), 71.81 (C-4‴), 68.24 (C-5‴), 17.73 (C-6‴). ESI-MS *m/z*=593 $[M–H]^-$.

Luteolin (7): A yellow powder (23 mg) (UV λ_{max}=267, 349 nm). ^{1}H-NMR (500 MHz, DMSO-d_6): *δ*12.98 (s, 5-OH), 7.42 (1H, dd, *J*=1.8, 8.3Hz, H-6'), 7.39 (1H, d, *J*=1.8Hz, H-2'), 6.88 (1H, d, *J*=8.3Hz, H-5'), 6.68 (1H, s, H-3), 6.44 (1H, d, *J*=2.0Hz, H-8), 6.19 (1H, d, *J*=2.0Hz, H-6). ESI-MS *m/z*= 285 $[M–H]^-$.

Luteolin-7-*O*-*β*-D-glucoside (8): A yellow powder (27 mg) (UV *λ*max=255, 268 sh, 348 nm). 1H-NMR (500 MHz, DMSO-d_6): δ 7.45(1H, dd, *J*=2.0,8.2Hz, H-6′), 7.43(1H, d, 2Hz, H-2′), 6.91 (1H, d, *J*=8.2Hz, H-5′), 6.79 (1H, d, *J*=2.2Hz, H-8), 6.74 (1H, s, H-3), 6.44 (1H, d, *J*=2.2Hz, H-6), 5.07 (1H, d, *J*= 7.5 Hz, H-1″). APT-NMR (125 MHz, DMSO-d_6): δ 164.6 (C-2), 103.5 (C-3), 182.3 (C-4), 161.6 (C-5), 99.9 (C-6), 163.4 (C-7), 95.2 (C-8), 157.4 (C-9), 105.8 (C-10), 121.7 (C-1′), 114.0 (C-2′), 146.3 (C-3′), 150.5 (C-4′), 116.5 (C-5′), 119.6 (C-6′), 100.3 (C-1″), 73.6 (C-2″), 76.8 (C-3″), 70.0 (C-4″), 77.7 (C-5″), 61.0 (C-6″). ESI-MS *m/z*=447 $[M–H]^-$.

Isorhamnetin (9): Ayellow amorphous powder (17 mg) (UV λ_{max}=255, 368 nm). ^{1}H-NMR (300 MHz, CD_3OD): δ 7.81 (1H, d, *J*=1.7Hz, H-2'), 7.68 (1H, dd, *J*=1.7, 8.4Hz, H-6'), 6.88(1H, d, *J*=8.4Hz, H-5'), 6.36 (1H, d, *J*=1.8Hz, H-8), 6.14 (1H, d, *J*=1.8 Hz, H-6), 3.89 (3H, s, H-3'). APT-NMR (75 MHz, DMSO-d_6): δ 146.60 (C-2), 135.78 (C-3), 175.85 (C-4), 160.62 (C-5), 98.18 (C-6), 163.65 (C-7), 93.58 (C-8), 156.09 (C-9),103.01 (C-10), 121.95 (C-1'), 111.72 (C-2'), 148.79 (C-3'), 147.35 (C-4'), 115.53 (CH-5'), 121.70(C-6'), 55.77 (OCH3). ESI-MS *m/z*= 315 $[M–H]^-$.

Gossypetin-3-*O*-*β*-D-rutinoside(10): A yellow amorphous powder (23 mg) (UV λ_{max}=262, 353 nm).^{1}H NMR (500 MHz, DMSO-d_6): δ 12.62 (1H,br s, 5-OH), 7.74 (1H, d, *J*=2.0Hz, H-2′), 7.65 (1H,dd, *J*=2&8.5Hz, H-6′), 6.81 (1H, d, *J*=8.5Hz, H-5′), 6.15 (1H, s, H-6), 5.39 (1H, d, *J*=7.7Hz, H-1″), 4.43 (1H, br. s, H-1‴), 1.03 (3H, d, *J*=6.2Hz, H-6‴). APT-NMR (125 MHz, DMSO-d_6): δ 156.19 (C-2), 133.07 (C-3), 177.76 (C-4), 158.86 (C-5), 99.80 (C-6), 153.89 (C-7), 122.8 (C-8), 148.4 (C-9), 104.80 (C-10), 121.60 (C-1′), 115.20 (C-2′), 144.73 (C-3′), 148.40 (C-4′), 116.45 (C-5′), 121.63 (C-6′), 101.54 (C-1″), 74.16 (C-2″), 76.44 (C-3″), 70.00 (C-4″), 75.92 (C-5″), 67.00 (C-6″), 100.70 (C-1‴), 70.32 (C-2‴), 70.57 (C-3‴), 71.86 (C-4‴), 68.29 (C-5‴), 17.78 (C-6‴). ESI-MS *m/z*=625 $[M–H]^-$.

Gossypetin-3-*O*-*β*-D-robinobioside [rhamnosyl (1→6) galactoside] (11): A yellow amorphous powder (22 mg) (UV λ_{max}=261, 352 nm). ^{1}H NMR (500 MHz, DMSO-d_6): δ 12.62 (1H, br.s, 5-OH), 7.75 (1H, d, *J*=2.0Hz, H-2′), 7.67 (1H, dd, *J*=2&8.5Hz, H-6′), 6.79 (1H, d, *J*=8.5Hz, H-5′), 6.14 (1H, s, H-6), 5.35 (1H, d, *J*=7.7Hz, H-1″), 4.45 (1H, br.s, H-1‴), 1.08 (3H, d, *J*=6.2Hz, H-6‴). ^{13}C APT-NMR (125.721 MHz, DMSO-d_6): δ 156.16 (C-2), 133.09 (C-3), 177.73 (C-4), 158.83 (C-5), 98.39 (C-6), 153.84 (C-7), 122.6 (C-8), 148.5 (C-9), 103.50 (C-10), 121.50 (C-1′), 115.10 (C-2′), 144.78 (C-3′), 148.50 (C-4′), 116.2 (C-5′), 121.65 (C-6′), 102.36 (C-1″), 71.20 (C-2″), 73.09 (C-3″), 67.95 (C-4″), 73.32 (C-5″), 64.79 (C-6″), 100.43 (C-1‴), 70.63 (C-2‴), 70.41 (C-3‴), 71.92 (C-4‴), 68.31 (C-5‴), 17.94 (C-6‴). ESI-MS/MS *m/z* = 625 $[M–H]^-$, and different fragments at *m/z* 317 $[M\text{-}H\text{-rhamnohexose}]^-$, 457 $[M\text{-}H\text{-rhamnohexose - }(1,3\ A_o\text{-}B_o)]^-$ and 489 $[M\text{-}H\text{- }(0,2\ A^+\text{-}B^+)]^-$.

***p*-Hydroxybenzoic acid (12):** Off-white solid (21 mg) (UV λmax=252 nm).^{1}H NMR (500 MHz, DMSO-d_6): δ 7.9 (d, *J*=8.5Hz, H-2,6), 6.8 (d, *J*=8.5Hz, H-3,5). APT-NMR (125 MHz, DMSO-d_6): δ 115.6 (C-3,5), 121.3 (C-1), 131.0 (C-2,6), 160.5 (C-4), 167.4 (COOH). ESI-MS *m/z*= 137 $[M–H]^-$.

Gallic acid (13): Acolorless crystal (18 mg) (UV λmax=272 nm).^{1}H-NMR (500 MHz, DMSO-d_6): δ 6.93 (2H, s). ESI-MS *m/z*= 169 $[M–H]^-$.

Brevifolin carboxylic acid (14): Yellow powder (16 mg) (UV $λ_{max}$= 277, 360 nm).^{1}H-NMR (400 MHz, DMSO-d_6): δ 7.15(s, H-7), 4.36 (d,*J*=6.2Hz,H-2), 2.95 (d, *J*=17.4Hz, H-3a), 2.54 (dd, *J*=6.2,17.4Hz, H-3b). ^{13}C-NMR (100 MHz, DMSO-d_6): δ 172.6(C-1), 42.3(C-2), 37.3(C-3), 195.1(C-4), 148.5(C-4a), 115.6(C-4b), 160.9(C-6), 112.7 (C-6a), 108.1(C-7),143.1(C-8), 140.9(C-9), 146.6(C-10), 140.1(C-10a). ESI-MS *m/z*=291 $[M–H]^-$.

Spectral Data of New Compound

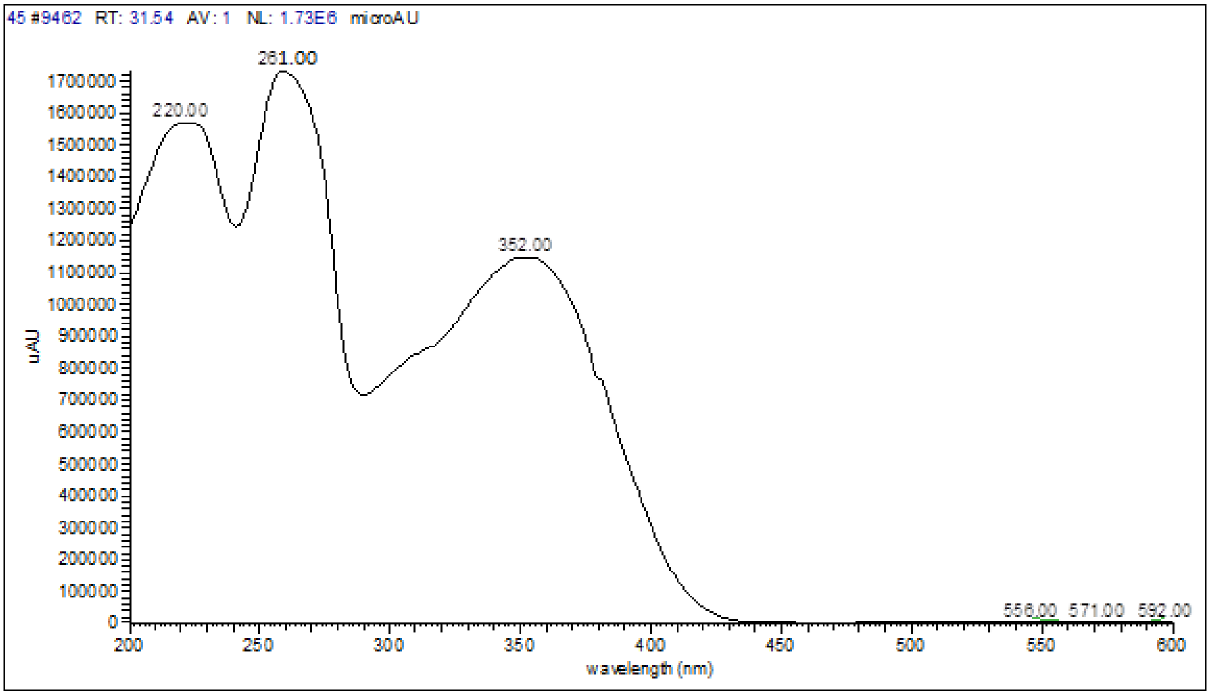

Fig. S1: UV spectrum of gossypetin-3-*O*-*β*-D-robinobioside [rhamnosyl (1→6) galactoside]

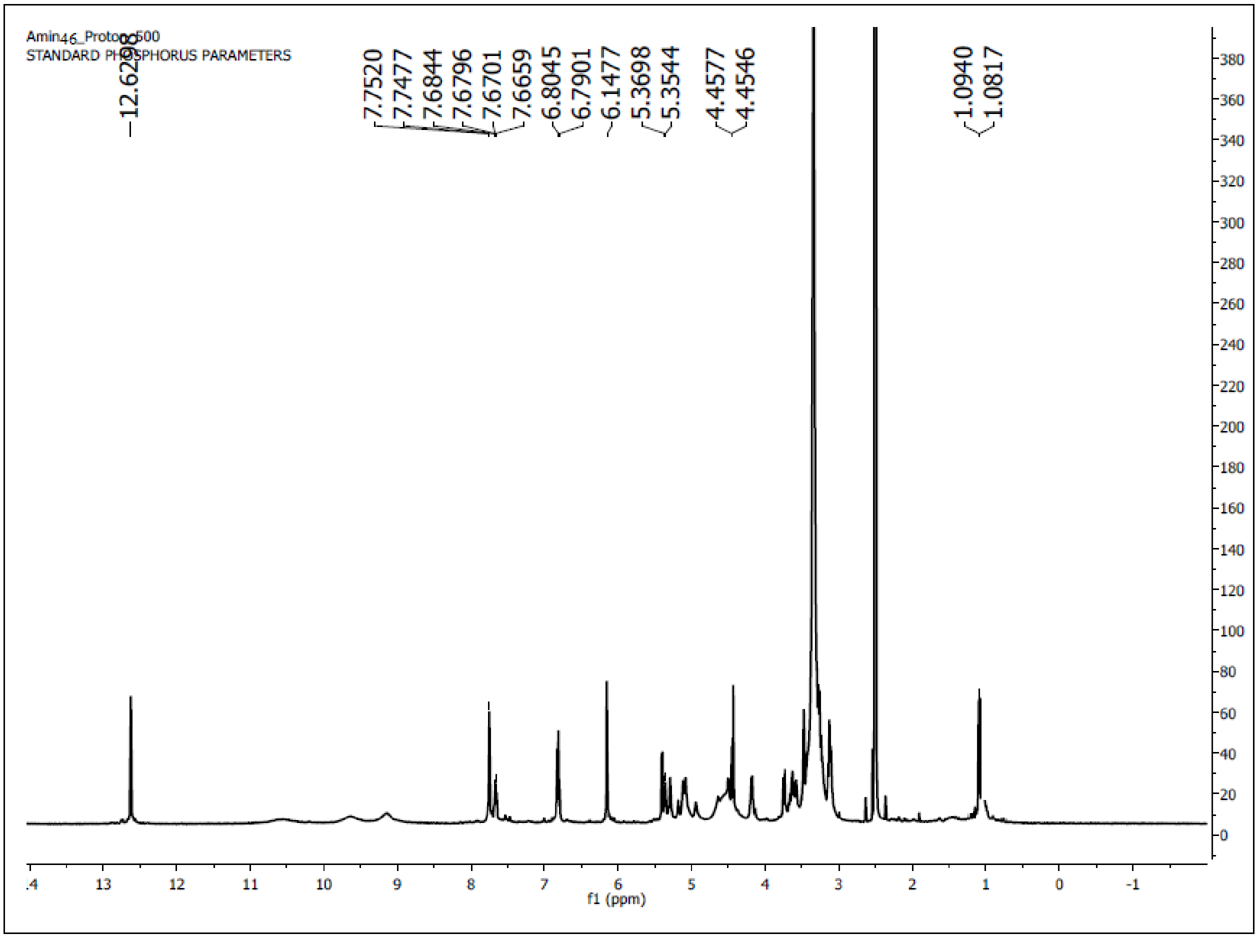

Fig. S2: ^{1}H-NMR spectrum of gossypetin-3-*O*-*β*-D-robinobioside [rhamnosyl (1→6) galactoside]

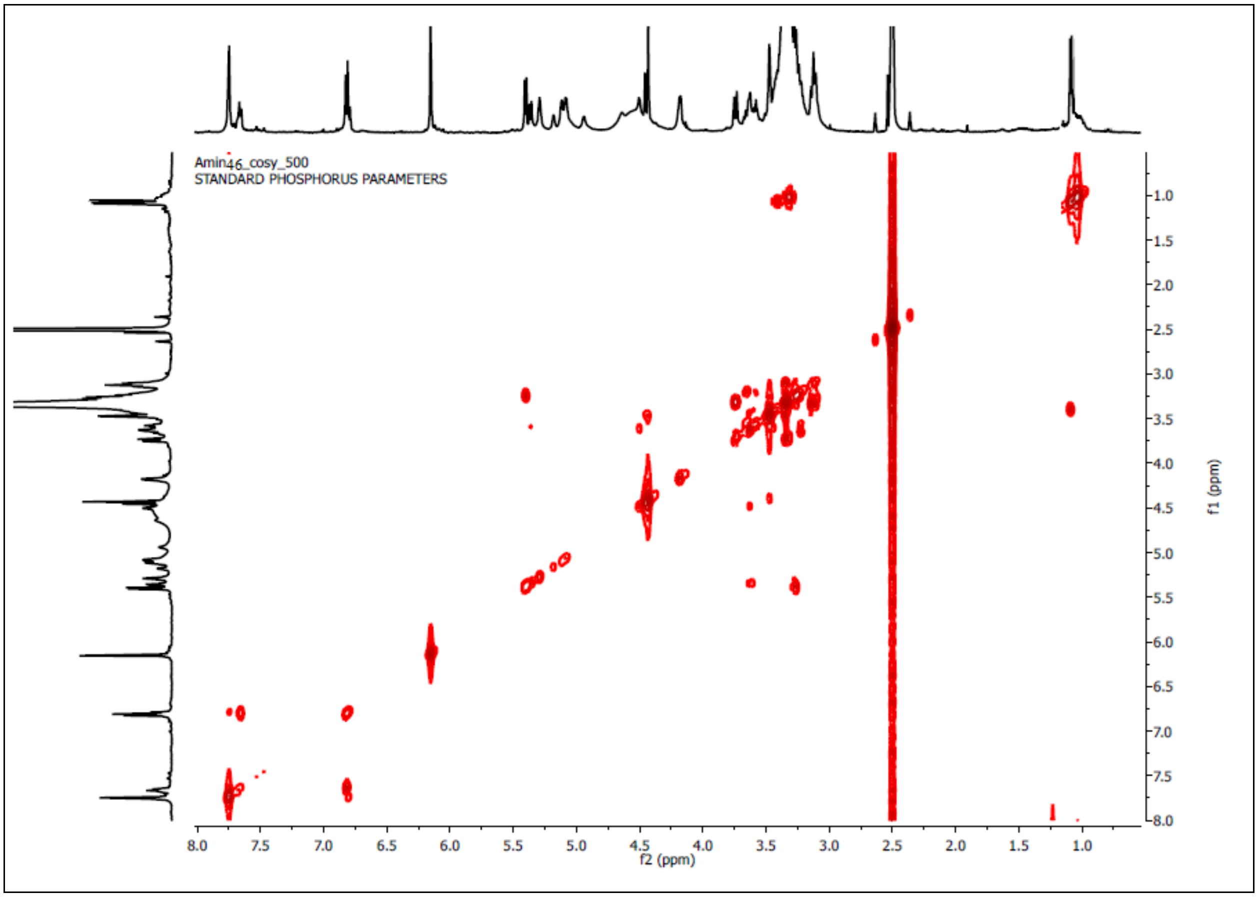

Fig. S3: HH-COSY NMR spectrum of gossypetin-3-*O*-*β*-D-robinobioside [rhamnosyl (1→6) galactoside]

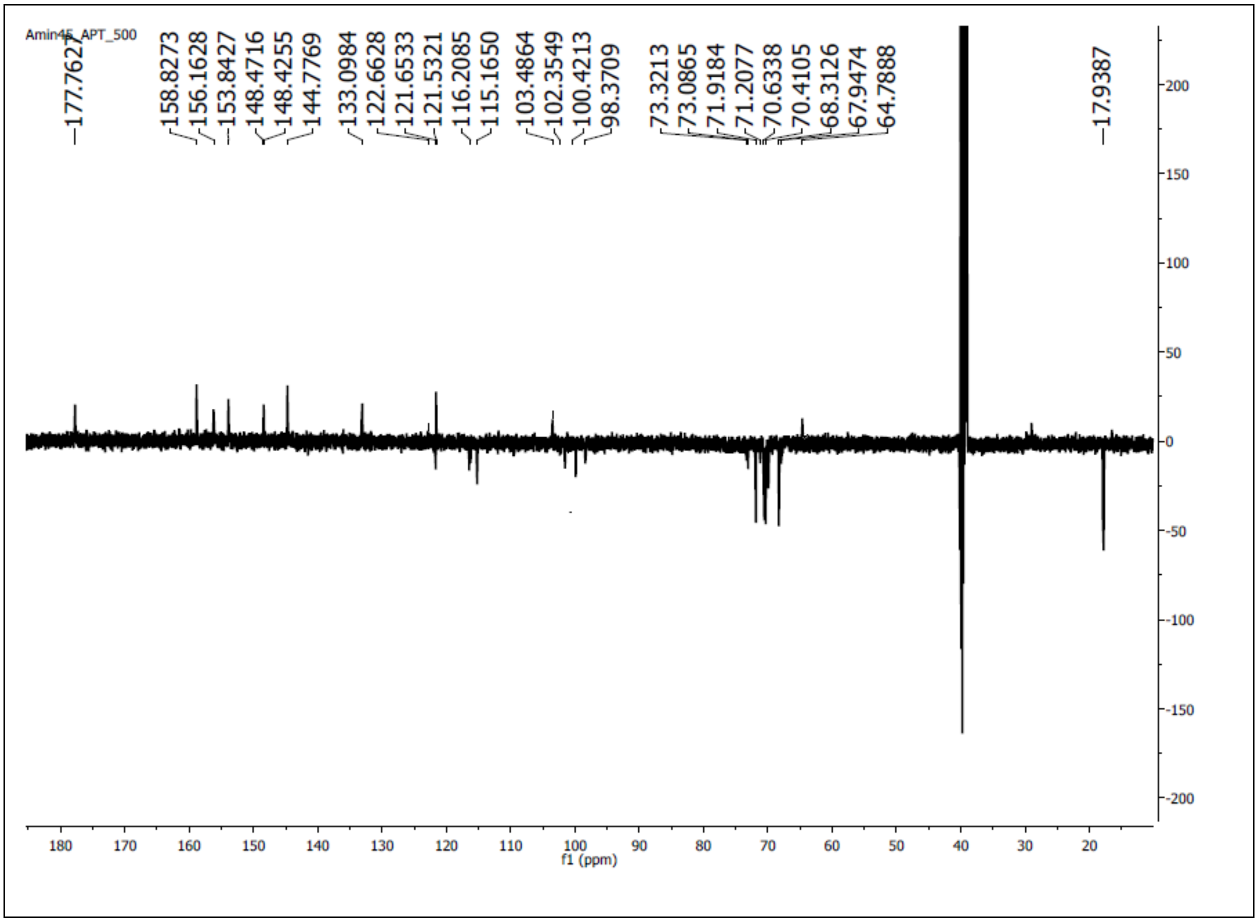

Fig. S4: APT-NMR spectrum of gossypetin-3-*O*-*β*-D-robinobioside [rhamnosyl (1→6) galactoside]

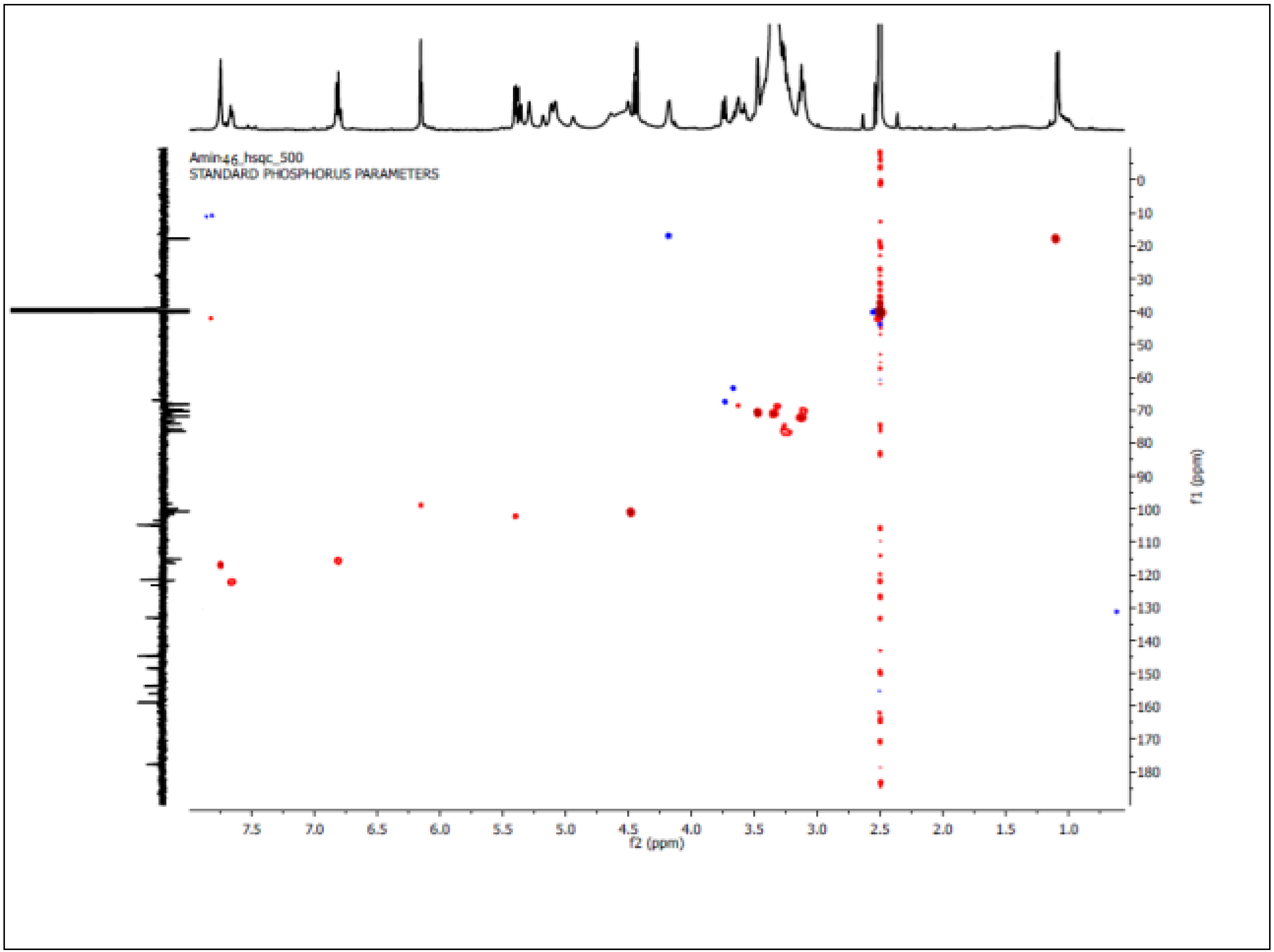

Fig. S5: HSQC-NMR spectrum of gossypetin-3-*O*-*β*-D-robinobioside [rhamnosyl (1→6) galactoside]

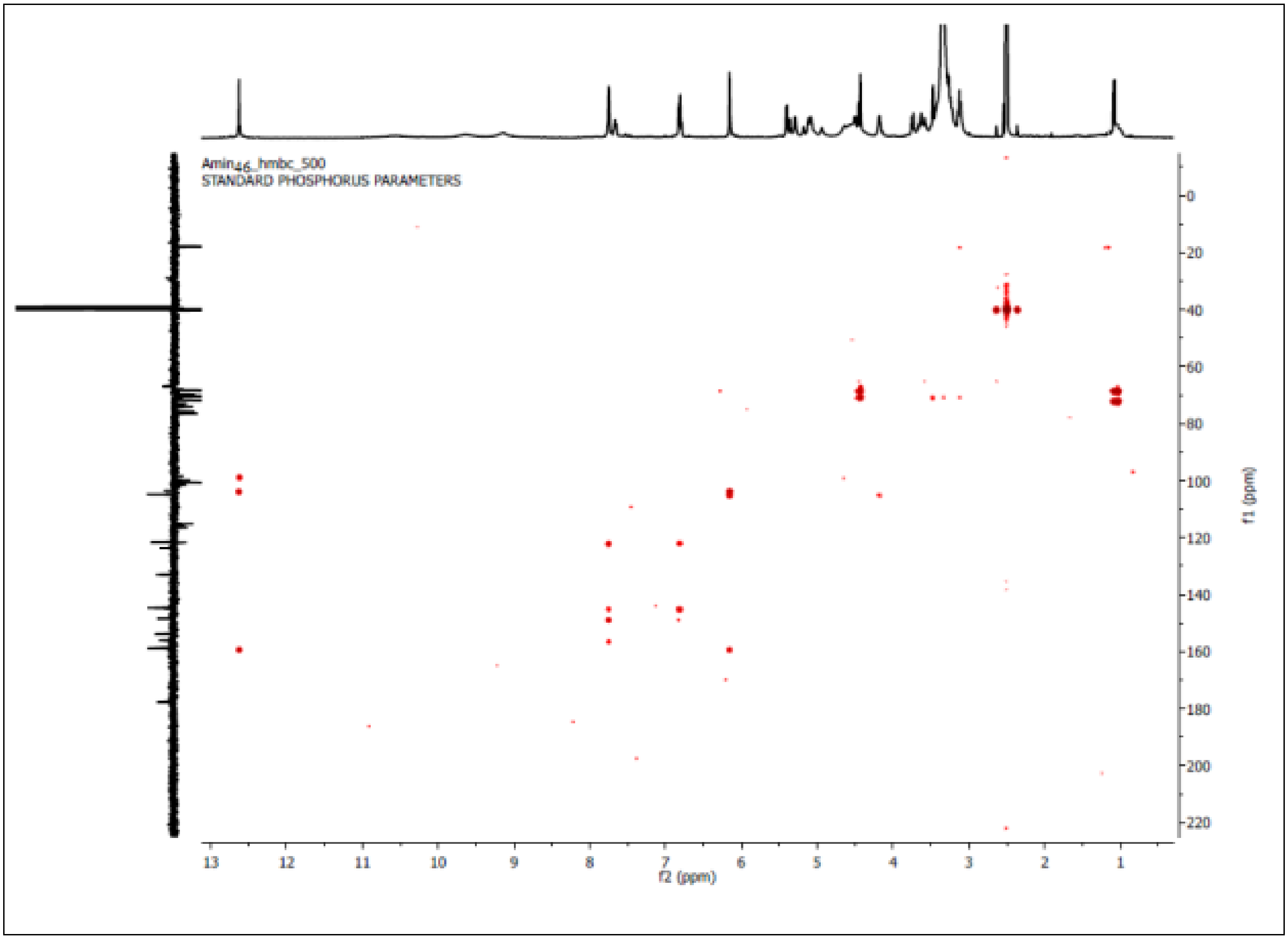

Fig. S6: HMBC-NMR spectrum of gossypetin-3-*O*-*β*-D-robinobioside [rhamnosyl (1→6) galactoside]

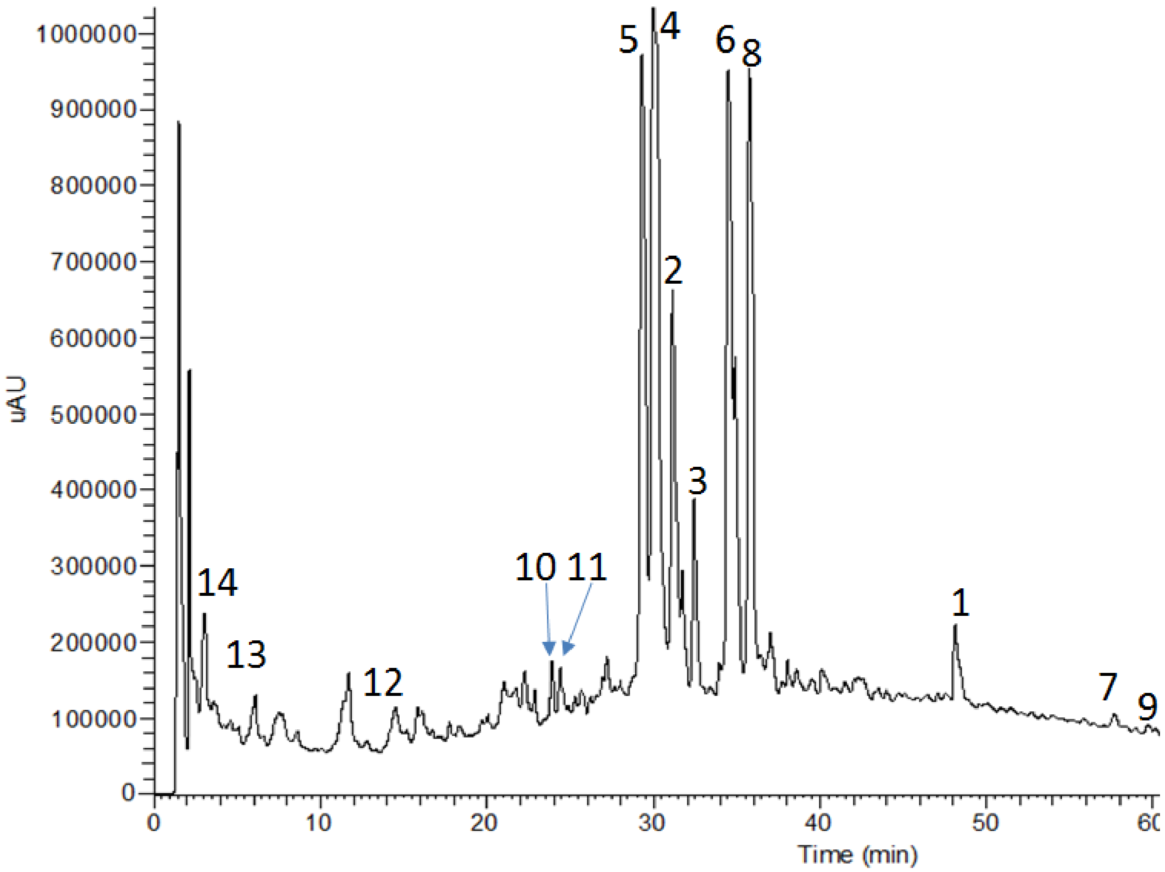

Fig. S7: HPLC–PDA profile of the methanol extract of of *Caesalpinia gilliesii*
